# Comparative Analysis of Robustness and Tracking Efficiency of Maximum Power Point in Photovoltaic Generators, Using Estimation of the Maximum Power Point Resistance by Irradiance Measurement Processing

**DOI:** 10.3390/s20247247

**Published:** 2020-12-17

**Authors:** Juan Ríos, Juan Manuel Enrique, Antonio Javier Barragán, José Manuel Andújar

**Affiliations:** Departamento de Ingeniería Electrónica, de Sistemas Informáticos y Automática, Universidad de Huelva, 21007 Huelva, Spain; juanm.enrique@diesia.uhu.es (J.M.E.); antonio.barragan@diesia.uhu.es (A.J.B.); andujar@uhu.es (J.M.A.)

**Keywords:** irradiance, measurement processing, maximum power point tracking, MPPT, maximum power point resistance, maximum power point tracking efficiency, DC/DC converter, duty cycle

## Abstract

The model-based methods of maximum power point (MPP) tracking in photovoltaic installations are widely known. One of these methods proposes the use of tracking by direct estimation of the maximum power point resistance using irradiance measurement processing. It proposes six different models for this estimate. In the present work, an exhaustive analysis to determine the robustness and accuracy of the different models was carried out. To perform the analysis, irradiance data sets, used to fit the parameters of the models, were collected. In addition, tests were done to determine MPP tracking accuracy of each of the six models. To carry out the tests, all models were compared with a widely used maximum power point tracking algorithm, *perturb & observe*, for different values of irradiance, temperature, and load.

## 1. Introduction

In order to increase the efficiency of photovoltaic facilities (to achieve maximum power transfer to the load), it is common to use maximum power point trackers (MPPT). Essentially, an MPPT is an impedance adapter (implemented by a DC/DC converter) connected between the photovoltaic generator (PVG) and the load [1,2]. This converter (its duty cycle) is governed by a control algorithm that attempts to bring the PVG to its maximum power point (MPP). It is well known that the MPP is not a static point, but is constantly fluctuating throughout the day, depending on the temperature and irradiance received by the PVG [1,2,3,4], hence the need to use effective MPPT. Compared to traditional tracking techniques [5,6,7,8], new ones have been developed, such as model-based techniques [9,10,11,12,13,14,15], and techniques based on artificial intelligence and bioinspired methods [2,3,16,17,18,19,20,21,22,23,24,25,26]. In general, these techniques seek to read the maximum power point without considering, at least a priori, the possibility that the installation works at a nonglobal maximum. For this reason, some techniques focus specifically on the search for the global maximum [4,16,17,19,21,23,24,25,26,27].

There are many proposals in the literature regarding the methods and control algorithms for MPPT, including works that try to classify and compare these methods [28,29,30,31,32,33]. However, in practice, the classical algorithm *perturb & observe* (P&O) [3,5,6,7,28] is the one most used due to its simplicity and easy implementation. Therefore, it is the best pattern with which to compare new proposals [28]. 

In [13], a new MPPT based on MPP resistance (*R_MPP_*) modeling was proposed. The key to this work is the ease in designing the MPPT, as the *R_MPP_* can be estimated only by knowing the incident solar irradiance. Although the results obtained were very accurate for virtually all six models, an MPP tracking efficiency analysis remained to be done for all of them. Additionally, the proposed MPPT still needed to be compared to the most commercially used MPPT, the P&O, to arrive at a practical outcome. 

This article aims to carry out the two aforementioned research activities. For this, different profiles of irradiance, temperature, and load have been considered. Likewise, the robustness of the different models was analyzed according to the set of data used in the process of identifying their parameters. The aforementioned work [13] lacks this robustness of analysis. 

This study is organized as follows. After the introduction, Section 2 presents the initial data and the DC/DC converter topology used for the analysis. In Section 3, the fitted models are determined based on experimental data. Section 4 is devoted to the comparison of the obtained efficiency between the different models regarding the P&O algorithm. For this, three days of sampled irradiance and temperature data, with different environmental conditions, were used. Naturally, different load profiles were also tested. The obtained results are discussed in Section 5. The paper ends with the main conclusions of this research.

## 2. Materials and Methods

**Figure 1:**
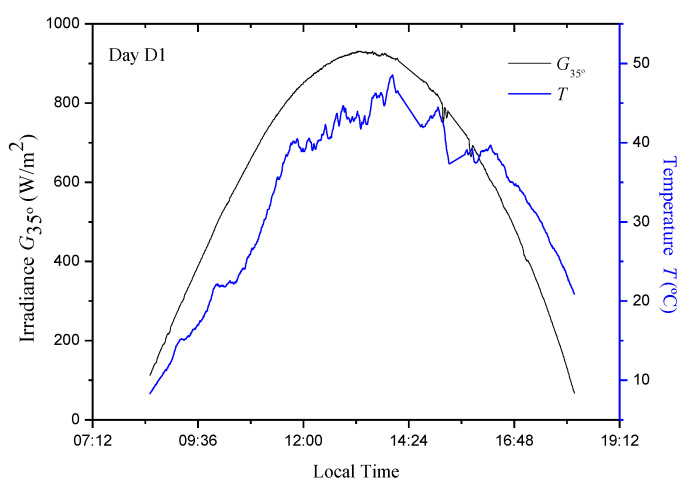
*T* and *G*_35°_ values in the photovoltaic generator (PVG) for D1 day.

**Figure 2:**
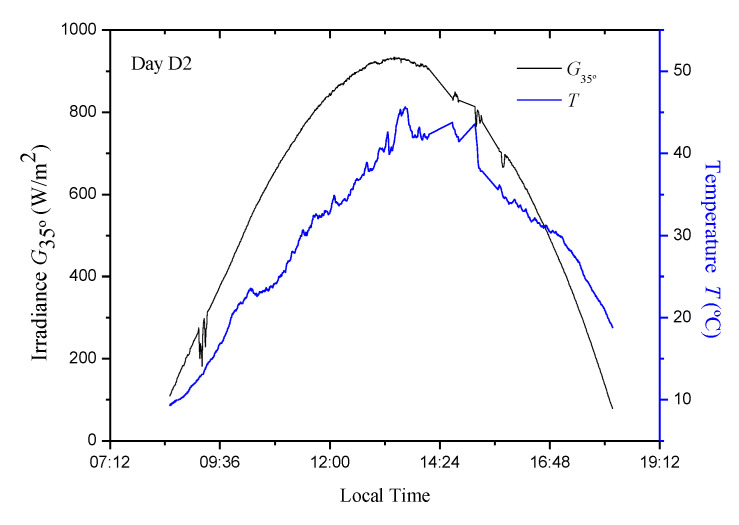
*T* and *G*_35°_ values in the PVG for D2 day.

**Figure 3:**
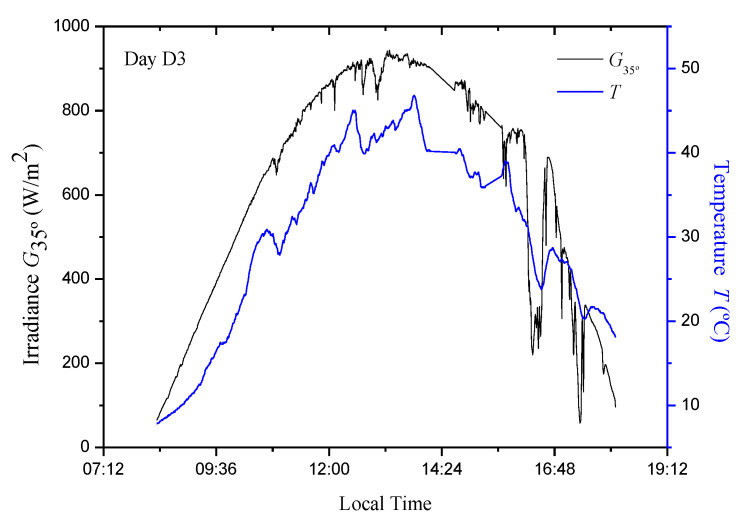
*T* and *G*_35°_ values in the PVG for D3 day.

For the current study, a data set containing different environmental conditions was used which corresponds to measures of irradiance values, (*G*) [34] and temperature, (*T*) [35,36] for three days (hereinafter called D1, D2, and D3). The measurements were made between 8:30 and 18:10 at 25 s intervals (Figure 1, Figure 2 and Figure 3). Naturally, these data are the same as those used in [13]. In the current study, an Isofoton^®^ ISF-250 PVG mounted at 35° (above the horizontal) was used, so that the irradiance measurement (W/m^2^) was made with that tendency (*G*_35°_). Simulations were carried out using the PVG model of a simple exponential and five parameters [1,2,3,16,37]. In regard to the PVG, its characteristics are summarized in Table 1.

**Figure 4:**
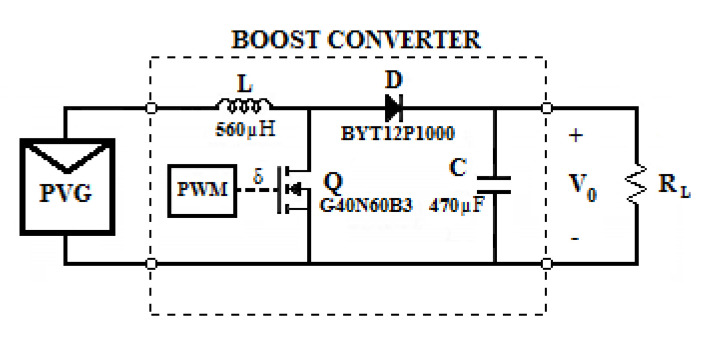
Boost *DC/DC* converter used in the simulations.

As per usual, for the simulations, it is acknowledged that the load is connected to the PVG by way of a boost converter (Figure 4).

As is well known, the PVG operates in its MPP when its *R_MPP_* matches the resistance that the DC/DC converter presents at its input [1,13].

The input resistance of the DC/DC converter can be calculated using Equation (1) [13,38]:

(1) $$R_{i}=R_{off}+R_{L}{\left(1-\delta \right)}^{2}$$

where *R_L_* is the load resistance and *δ* is the converter duty cycle. *R_off_* is a resistance that includes an inductor, connection wires between the PVG and the DC/DC converter, and other parasitic resistances of the converter. For this study, a practical *R_off_* = 2 Ω value was taken. This value was obtained experimentally as detailed in [13]. Thus, when the system operates in the MPP, Equation (1) can be rewritten as

(2) $$R_{MPP}=R_{off}+R_{L}{\left(1-\delta_{opt}\right)}^{2}$$

where *δ_opt_* is the optimum duty cycle that makes the PVG work at its MPP, which can be easily derived from Equation (2):

(3) $$\delta_{opt}=1-\sqrt{\frac{R_{MPP}-R_{off}}{R_{L}}}.$$

## 3. *R_MPP_* Modeling

**Figure 5:**
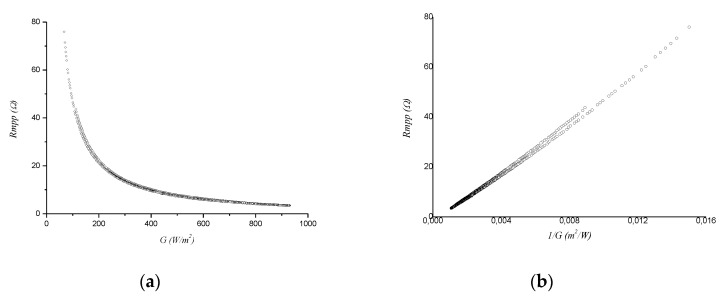
(**a**) *R_MPP_-G* and (**b**) *R_MPP_-1/G* characteristics for the module ISF-250.

Following the method described in [13], the PVG was characterized at its MPP. From here, *R_MPP_* versus G_35°_ and 1/G_35°_ (Figure 5a,b, respectively) can be drawn. These were performed using data from D1, however, no significant differences were found when using data from other days.

Equations (4)–(10) show the six models used to approximate the *R_MPP_* characteristics [13]. Note that it is only necessary to have irradiance measurements.

### 3.1. Exponential Model

It is a natural proposal based on the curve shape *R_MPP_* versus *G*_35°_.

(4) $$R_{MPP-exp}=A_{1}+B_{1}\times e^{\frac{-G_{35{}^{\circ}}}{C_{1}}}.$$

### 3.2. Hyperbolic Model

It is a natural proposal based on the curve shape *R_MPP_* versus 1/*G*_35°_.

(5) $$R_{MPP-hyp}=A_{2}+\frac{B_{2}}{G_{35{}^{\circ}}}.$$

### 3.3. Polynomial Order 2 Model

Another classical modeling mode is to use a polynomial regression by an *n*th degree polynomial in 1/*G*_35°_, in this case; thus, *n* = 2,

(6) $$R_{MPP-pol2}=A_{3}+\frac{B_{3}}{G_{35{}^{\circ}}}+\frac{C_{3}}{G_{35{}^{\circ}}{}^{2}}.$$

### 3.4. Polynomial Order 3 Model

As in the previous model, but now with *n* = 3.

(7) $$R_{MPP-pol3}=A_{4}+\frac{B_{4}}{G_{35{}^{\circ}}}+\frac{C_{4}}{G_{35{}^{\circ}}{}^{2}}+\frac{D_{4}}{G_{35{}^{\circ}}{}^{3}}.$$

### 3.5. Weighted Model

If the real *R_MPP_* and the exponential and hyperbolic models are represented in the same graph, it becomes apparent that there is an opposing variance at the ends of the models, and therefore, suggests a combination of models, Equations (4) and (5), in order to cancel the opposite deviations that both have at the ends, i.e.,

(8) $$R_{MPP-weig}=X_{5}\times R_{MPP-exp}+\left(1-X_{5}\right)\times R_{MPP-hyp}\;.$$

Thus,

(9) $$R_{MPP-weig}=X_{5}\times \left(\;A_{1}+B_{1}\times e^{\frac{-G_{35{}^{\circ}}}{C_{1}}}\right)+\left(1-X_{5}\right)\times \left(A_{2}+\frac{B_{2}}{G_{35{}^{\circ}}}\right).$$

### 3.6. OEH Model (Offset, Exponential, and Hyperbolic)

(10) $$R_{MPP-oeh}=A_{6}+B_{6}\times e^{\frac{-G_{35{}^{\circ}}}{C_{6}}}+\frac{D_{6}}{G_{35{}^{\circ}}}.$$

In addition to the smaller number of parameters, the difference between Equations (9) and (10) is that in the latter case, the set of parameters was set by the researchers.

For models fitting estimation of parameters A, B, C, D, and X, the least squares method was used, obtaining three sets of parameters corresponding to each of the three days (D1, D2, and D3). Table 2 shows the obtained parameters, as well as the root mean square $\left(RMSE=\sqrt{\frac{1}{N}\overset{N}{\underset{i=1}{\sum }}{\left(y_{i}-\hat{y_{i}}\right)}^{2}}\right)$ and the normalized mean absolute error $\left(NMAE=\frac{1}{N}\overset{N}{\underset{i=1}{\sum }}\left|y_{i}-\hat{y_{i}}\right|\right)$ of the models; with *y_i_* representing the observed value (measured), *N* the numbers of measurements carried out, and $\hat{y_{i}}$ the estimated measurement by the model. 

**Figure 6:**
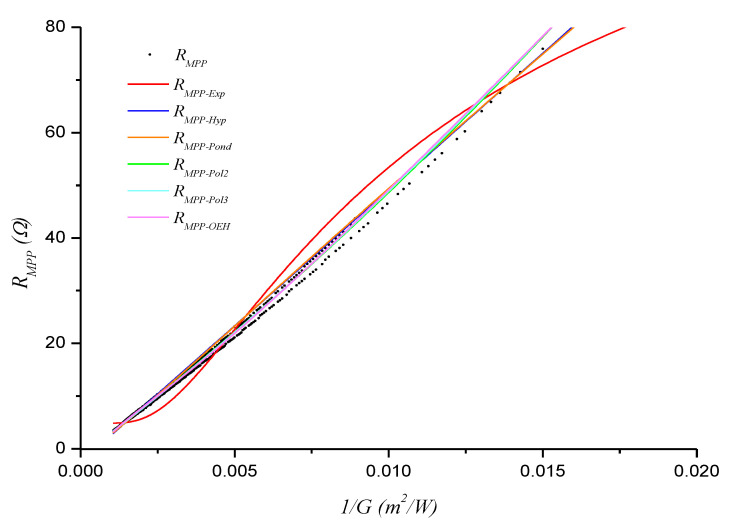
Functions fitted with the D1 data.

**Figure 7:**
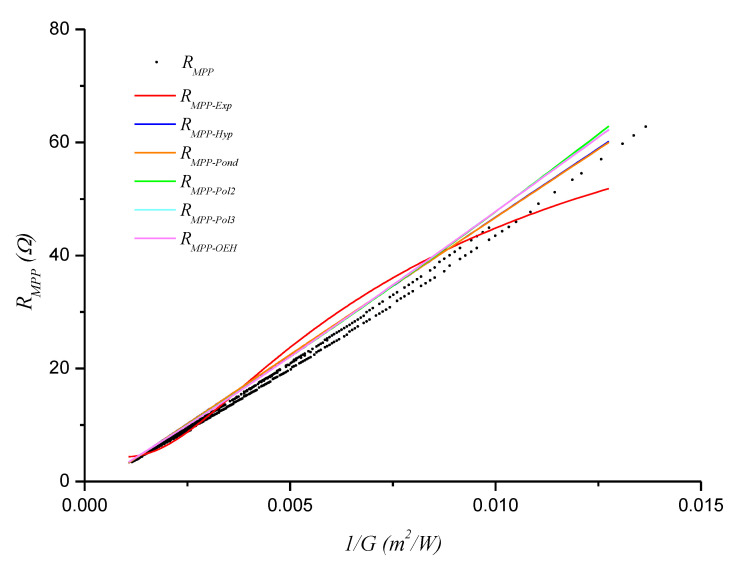
Functions fitted with the D2 data.

**Figure 8:**
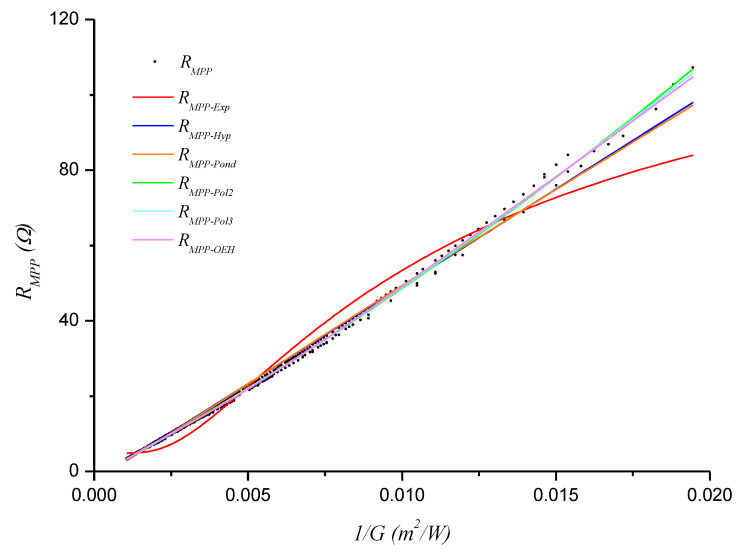
Functions fitted with the D3 data.

Figure 6, Figure 7 and Figure 8 show the obtained *R_MPP_* versus 1/*G* models (functions) for days D1, D2, and D3, respectively.

## 4. Results

In this section, the evaluation and comparison of the proposed models with the P&O algorithm are carried out. As there are three sets of parameters for each model, the evaluation was done through a cross validation procedure, which consists of evaluating the efficiency of each model using data from the days that were not used for its adjustment. From the mathematical model of the photovoltaic facilities (PVG + DC/DC converter + Load) and measured irradiance and temperature data (Figure 1, Figure 2 and Figure 3), it is possible to calculate the following: (i) the actual MPP power (*P_MPP_*); (ii) the instantaneous power delivered by the P&O MPPT; and (iii) the instantaneous powers delivered by each one of the models (4), (5), (6), (7), (9), and (10), which is obtained by determining the optimum duty cycle (*δ_opt_*) of the DC/DC converter (3).

The parameter that measures the goodness of an MPPT is the performance or tracking efficiency, *η*, Equation (11) [28]:

(11) $$\eta =\frac{\int_{0}^{t}P_{inst}\left(t\right)dt}{\int_{0}^{t}P_{MPP}\left(t\right)dt}\;$$

where *P_inst_*(*t*) is the power supplied by the PVG controlled by the MPPT under study (modeling) and *P_MPP_*(*t*) the power of the actual MPP for the irradiance and temperature conditions given at the evaluated sample time.

From here, the comparison of all the proposed MPPTs regarding P&O are carried out. First, a constant load should be considered, followed by a variable load, even with a high rate of change, both in terms of amplitude and speed.

### 4.1. Simulation with Constant Load

The constant load was chosen depending on the DC/DC converter characteristics; in this case, *R_L_* = 55 Ω (see Figure 4). The performance of the fitted model for one day was evaluated with regard to other days. Table 3 shows the obtained performances of the P&O MPPT and those obtained using each of the proposed models.

### 4.2. Simulation with Variable Load

**Figure 9:**
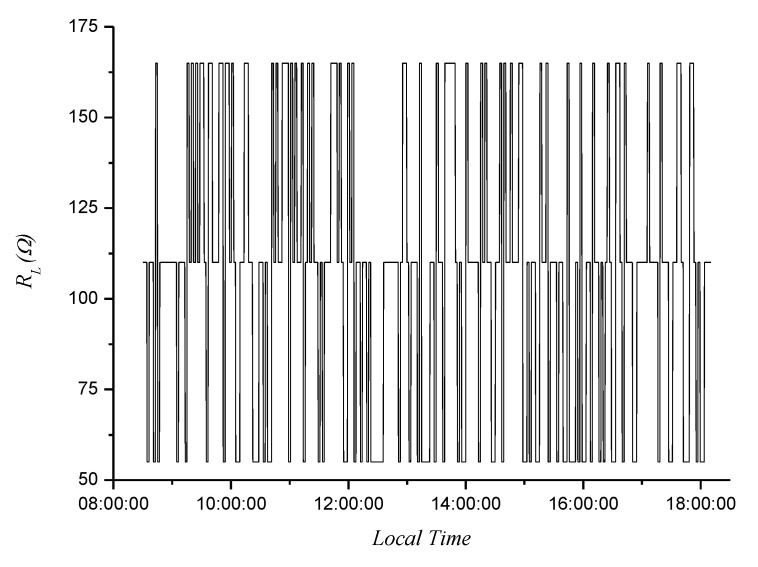
Variable load profile.

In this test, the PVG was supplied to a load through the DC/DC converter. The load varied approximately every 2 min, randomly taking values of *R_L_*, 2 *R_L_*, and 3 *R_L_*, with *R_L_* = 55 Ω, i.e., 55, 110, and 165 Ω. Figure 9 shows the load profile.

The performances obtained by the P&O MPPT, and those obtained using each of the proposed models, are summarized in Table 4.

### 4.3. Simulation with Greater Amplitude of Variation in the Load

A complementary test was carried out in which the PVG supplied to a load that varied more widely than the previous case. Specifically, *R_L_* took the values 25 Ω, 150 Ω, and 275 Ω with a change period of 2 min. As this test was like the previous one, its only purpose was to demonstrate that this test was carried out only in one of the cases. Table 5 shows the results.

### 4.4. Change Frequency Dependence

To evaluate the models, in terms of the speed of the load changes, a final test was carried out. Now, *R_L_* can take randomly 55 Ω, 110 Ω, and 165 Ω values, with periods of change at 1, 5, 10, and 20 min, approximately. D2 was the model evaluation day and D1 was the model fitting day. Table 6 shows the obtained performances.

## 5. Discussion

The results obtained in terms of robustness and tracking efficiency are shown in Table 2, Table 3, Table 4, Table 5 and Table 6. In these tables, except in Table 2, the results obtained using the classic P&O algorithm are also included. From the analysis of these results, it can be inferred that:

- The exponential model had the poorest performance. It had a poor fit over the experimental data, with a relatively high mean square error, 4.45 Ω, around an order of magnitude higher than the rest of the models (Table 2). When comparing the results in terms of tracking efficiency, using this model against the P&O algorithm, worse results were obtained when working with a constant load (Table 3). When working with a variable load, the performance of the exponential model remained practically invariable (around 90%); however, the P&O algorithm presented a considerable loss of performance, especially as the variability of the load increased (Table 4, Table 5 and Table 6);
- The other models performed better than the P&O algorithm, both with constant and variable loads. However, the hyperbolic and weighted models showed certain dependencies with the data used for their fitting (Table 2). The polynomial models and the OEH model presented the best results in terms of tracking efficiency (Table 4) and a great independence or robustness, both for the data (days) used for their fitting, and of the yet to be evaluated environmental characteristics of the day. These conclusions were obtained from the analysis of the data in Table 2 for function fitting data, and in Table 3 and Table 4 for efficiency results;
- The differences between the best models and the P&O algorithm became more evident when the system operated with variable load. If the difference in performance with fixed load was around 4% in favor of the models, this difference elevated to 10%, and almost 20% in the tests carried out with variable and highly variable loads, respectively. It was observed that the performance of the P&O algorithm had a high dependence on the variations of the load, while the *R_MPP_* estimating models were immune to this circumstance. Table 4 and Table 5 support this conclusion;
- It can also be observed that the performance of the P&O algorithm, in the case of variable load, depended on the frequency of change of the load, receiving worse results as the frequency of change increased. Again, the models were shown to be immune to this circumstance (Table 6).

## 6. Conclusions

In this work, an exhaustive analysis was carried out on the method for tracking the maximum power point (MPP) of a photovoltaic generator by direct estimation of the MPP resistance, *R_MPP_*. Six functions were obtained that model the *R_MPP_* of a photovoltaic generator as a function of the radiation received. For this purpose, the real radiation and temperature data measured on three days with different environmental characteristics were used. These functions were used to implement the maximum power point tracking method by direct estimation of *R_MPP,_* evaluating the performance achieved with each of them, and, in turn, comparing it with that obtained by the classic and probably most popular method, *Perturb & Observed* (P&O). At the same time, an analysis of the robustness of this new method was also carried out, evaluating the results obtained by the different models when these were adjusted with data obtained on different days, with different environmental conditions. From the analysis of the obtained results the following conclusions can be derived:

- It appears that the use of models for the estimation of *R_MPP_* and calculation of the duty cycle of the converter is an interesting alternative, especially in systems where the load presents variations;
- All the models studied presented better performances than the P&O algorithm, except for the exponential model, which in the case of fixed loads did not present better data than P&O. Nevertheless, thus far, the system has been subjected to variable loads, and this model has presented great benefits;
- Of the six studied models, the polynomial of order 2 presents the most interesting alternative. It presents a robust result, regardless of the data used for its fitting and the characteristics of the evaluation day. Its performance shows no dependence on variations in the system load or on its frequency of variation, reaching values above 99%, in all cases. In addition, it is the model that has the fewest parameters, and therefore the easiest to adjust among the models that have these good characteristics;
- With regard to the performance of the method, it can be concluded that it presents a good behavior, both in the transient (search for the MPP) and in the steady state (very slow changes). Some of the model-based methods referenced in this work obtain a good dynamic behavior but a behavior that can be improved in the steady state.

## Figures and Tables

**Table 1:** Parameters of the photovoltaic module ISF-250.

A = 1.2	Ideality factor of PN junction
Eg = 1.12 eV	Band gap energy
n_p_ = 6	Number of parallel-connected modules
n_s_ = 10	Numbers of series-connected cells
P_max_ = 250 W	Maximum power at standard conditions *
V_max_ = 30.6 V	Voltage at the maximum power point
I_max_ = 8.17 A	Current at the maximum power point
NOTC = 45 °C	Nominal Operating Cell Temperature
I_sc_ = 8.75 A	Short-circuit current at standard conditions *
V_oc_ = 37.8 V	Open-circuit voltage at standard conditions *
K_v_ = −0.323%/°C	V_oc_ temperature coefficient
K_i_ = 0.042%/°C	I_sc_ temperature coefficient
R_s_ = 0.82 Ω	Series resistance
R_p_ = 324 Ω	Parallel resistance

* Standard conditions: 25 °C and 1000 W/m^2^.

**Table 2:** Obtained parameters and *R_MPP_* fitting errors.

Model	Parameters	Day 1	Day 2	Day 3
Exponential	A_1_ (Ω)	4.4022	4.2984	4.9074
	B_1_ (Ω)	93.4369	84.6109	132.8013
	C_1_ (W/m^2^)	126.4264	135.9575	99.3839
	**RMSE (Ω)**	**1.8418**	**1.2149**	**4.4477**
	**NMAE (%)**	**14.87**	**12.59**	**20.92**
Hyperbolic	A_2_ (Ω)	−2.0130	−1.9144	−2.4976
	B_2_ (Ω W/m^2^)	4879.1	4868.5	5171.7
	**RMSE (Ω)**	**0.2113**	**0.1357**	**0.6772**
	**NMAE (%)**	**3.64**	**2.89**	**6.18**
Polinomial order 2	A_3_ (Ω)	−1.4179	−1.3827	−1.3513
	B_3_ (Ω W/m^2^)	4442.3	4463.0	4411.4
	C_3_ (Ω W^2^/m^4^)	44,379	45,112	59,517
	**RMSE (Ω)**	**0.1209**	**0.0790**	**0.1589**
	**NMAE (%)**	**2.15**	**2.05**	**1.45**
Polinomial order 3	A_4_ (Ω)	−1.3453	−1.2062	−1.1696
	B_4_ (Ω W/m^2^)	4371	4300	4247.4
	C_4_ (Ω W^2^/m^4^)	59,800	88,000	89,000
	D_4_ (Ω W^3^/m^6^)	−826,110	−2,617,800	−1,260,000
	**RMSE (Ω)**	**0.1204**	**0.0771**	**0.1537**
	**NMAE (%)**	**2.14**	**2.06**	**1.66**
Weighted	A_1_ (Ω)	4.4022	4.2984	4.9074
	B_1_ (Ω)	93.4369	84.6109	132.8013
	C_1_ (W/m^2^)	126.4264	135.9575	99.3839
	A_2_ (Ω)	−2.0130	−1.9144	−2.4976
	B_2_ (Ω W/m^2^)	4879.1	4868.5	5171.7
	X_5_	0.0085	0.0210	0.0547
	**RMSE (Ω)**	**0.2111**	**0.1352**	**0.6646**
	**NMAE (%)**	**3.54**	**2.70**	**5.07**
OEH	A_6_ (Ω)	−2.2327	−2.0655	−3.0792
	B_6_ (Ω)	−6.3205	−5.1896	−14.8579
	C_6_ (W/m^2^)	268.3028	293.0880	209.2151
	D_6_ (Ω W/m^2^)	5424.1	5353.1	6148.9
	**RMSE (Ω)**	**0.1214**	**0.0765**	**0.1551**
	**NMAE (%)**	**2.16**	**2.05**	**1.69**

**Table 3:** Obtained performances (percentage of tracking efficiency) with constant load *R_L_* = 55 Ω.

Model Evaluation Day	Model Fitting Day	Models						
		P&O	Expo.	Hyp.	Pol. 2	Pol. 3	Pond	EOH
1	2	96.93	92.86	99.31	99.56	99.44	99.41	99.53
1	3		87.67	96.14	99.57	99.49	97.43	99.48
2	1	96.79	91.86	97.78	99.30	99.33	97.87	99.36
2	3		88.10	95.39	99.58	99.59	96.64	99.38
3	1	95.34	92.37	98.19	99.60	99.61	98.29	99.64
3	2		93.39	99.31	99.78	99.72	99.43	99.76

**Table 4:** Obtained performances (percentage of tracking efficiency) with variable load.

Model Evaluation Day	Model Fitting Day	Models						
		P&O	Expo.	Hyp.	Pol. 2	Pol. 3	Pond	EOH
1	2	89.05	92.86	99.32	99.56	99.45	99.41	99.54
1	3		87.67	96.14	99.57	99.49	97.43	99.48
2	1	90.16	91.86	97.78	99.30	99.33	97.87	99.36
2	3		88.10	95.39	99.58	99.59	96.64	99.38
3	1	88.92	92.37	98.20	99.61	99.62	98.30	99.66
3	2		93.39	99.32	99.80	99.74	99.44	99.78

**Table 5:** Obtained performances (percentage of tracking efficiency) with highly variable load.

Model Evaluation Day	Model Fitting Day	Models						
		P&O	Expo.	Hyp.	Pol. 2	Pol. 3	Pond	EOH
3	2	81.08	93.33	99.26	99.74	99.68	99.38	99.72

**Table 6:** Obtained performances (percentage of tracking efficiency) with different load change frequencies.

Model Evaluation Day	Model Fitting Day	Change Period	Models						
			P&O	Expo.	Hyp.	Pol. 2	Pol. 3	Pond	EOH
2	1	1 min	86.92	91.86	97.77	99.29	99.33	97.87	99.36
2	1	5 min	92.34	91.86	97.77	99.29	99.33	97.87	99.36
2	1	10 min	92.90	91.86	97.77	99.29	99.33	97.87	99.36
2	1	20 min	94.63	91.86	97.77	99.29	99.33	97.87	99.36
